# Vulnerability and resilience of the road transport industry in Poland to the COVID-19 pandemic crisis

**DOI:** 10.1007/s11116-021-10246-9

**Published:** 2021-12-02

**Authors:** Magdalena Osińska, Wojciech Zalewski

**Affiliations:** Faculty of Economic Sciences and Management, Nicolaus Copernicus University in Toruń, Gagarina 13 A Street, 87-100, Toruń, Poland

**Keywords:** Creative destruction, Microdata, Demand shock, Profitability, Bankruptcy, Logit model

## Abstract

The research aims to examine the vulnerability and resilience of road transport enterprises in Poland to a crisis caused by the COVID-19 pandemic. In theory, we refer to the Schumpeterian perspective of creative destruction. In the empirical analysis, survey data on 500 transport companies randomly selected from the database were used. We estimated partial proportional odds models to show the factors responsible for the enterprises' vulnerability and resilience to unforeseen shock. The perspective refers to the total sample size and the division into two subgroups: micro and small and medium enterprises. To justify the results, we calculated a set of statistical indicators and tests. These models enable separating enterprises according to the vulnerability level. Transport enterprises occurred significantly vulnerable to the COVID-19 crisis, particularly the demand shock. The only factor that influenced resilience was the decrease in fuel prices, which allowed a cost reduction. The crisis showed that government aid was helpful in the short run, particularly for micro and small enterprises. The medium-sized enterprises were more resilient than micro and small ones. We formulated several recommendations to help transport enterprises to adjust in the medium term.

## Introduction

The study focuses on the impact of the crisis caused by the first phase of the COVID-19 pandemic on the economic and financial performance of the road transport industry from the representative enterprises' perspective. The COVID-19 pandemic and the lockdown announced by the governments was a real shock for all spheres of economic and social life. As a consequence, it caused a severe crisis which results are still not fully recognized. However, it has not been the only crisis that has affected the transportation sector since the beginning of the XXI century.

In the last two decades, the transport industry has faced three acute crises. These were: the terrorist attack on World Trade Centre (WTC) on September 11, 2001, the global financial and economic recession in the years 2007–2009, and the COVID-19 pandemic, which has been observed since December 2019. Looking at the consequences of these crises for transport enterprises, we should start with the first one related to the WTC terrorist attack on September 11, 2001, and those that followed it in Madrid in 2004 and London in 2005. The 9/11 shock caused changes, mainly in air transportation, which managed to recover only in 2004 (Notis 2005). The consequences for road transport included trucks and load tracking, supported by digital technology's rapid development and immediate access to GPS/GPRS systems. The increase in global trade and the growing threat has forced customs administrations worldwide to redirect their attention away from the traditional tasks of controlling shipments and collecting customs data to a greater focus on the security of the international exchange of goods. Their results are several projects and security arrangements, such as the SAFE *Framework* of *Standards* of 2005 and 2018 provided by the World Customs Organization. (SAFE 2005; SAFE 2018).

In 2007–2009, the financial crisis, which caused a global economic recession, took place. It resulted from the loose monetary policy followed by subprime mortgages, weak regulatory structures, and high leverage in the banking sector (Allen and Carletti 2010). It severely touched the world economy, including transport enterprises. Rothengatter (2011) analyzed the impact of the recession on the transport sector. He took the perspective of continuous innovation and creative destruction*,* as Schumpeter defined (Freeman 2009). In this meaning, the crisis is considered a constructive structural break if it results in innovations that change business. However, if firms do not adjust their strategies to the conditions changed by possible discontinuity and gradually develop their activity in a continuous-time path, their future is rather pessimistic (Foster and Kaplan 2001). Rothengatter (2011) pointed out that the economic crisis in 2007–2009 has led to a dramatic reduction in international trade and freight transport in several European countries. He did not consider Polish road transport enterprises, which – apart from common difficulties—were the only example in Europe that managed to increase their total value of transport in 2009 compared to 2008 by 9.6% (Wrzesińska 2011). As Rottengatter noticed, transport firms adjusted through short-term contracting and lean inventory holding to save costs in the short run and foster flexibility in a phase of high uncertainty. However, the results of the crisis for transportation were also positive when the medium run is considered. Due to this structural break, transport operations started to become more energy-efficient and environmentally friendly. These mean emissions reduction in truck construction and reduction in the road transport industry favor intermodal transport.

The COVID-19 pandemic broke out in December 2019. By January 8, 2021, there had been reported 89.7 million COVID-19 cases, and the death toll exceeded 1.9 million cases worldwide (Worldometers, 2021). The COVID-19 pandemic caused a general social and economic lockdown and has substantial economic consequences on both a micro-and macro-scale, which have been visible particularly since March 2020. It became a severe discontinuity, causing a massive challenge for the economy as a whole and the transport industry in particular. On the macro-scale, Poland's GDP growth in the second quarter of 2020 was reduced by 8.2% compared to the same period in 2019. The Euro area and the European Union were much worse, since the GDP decrease amounted to 15.0% and 14.1%, respectively, compared with the same quarter of the previous year (Eurostat 2020). The services sector, due to the close contact with customers, was practically closed or strongly limited. Ozili and Arun (2020) explained how the coronavirus outbreak led to spillovers into significant sectors of the global economy and how several governments' fast policy responses triggered or prolonged the recession while trying to save citizens' lives. Gössling et al. (2021) analyzed the impact of the current and some previous epidemics on tourism. The authors explore the COVID-19 crisis as an opportunity to reconsider tourism’s growth trajectory critically and to question the logic of more arrivals implying greater benefits. This idea can be generalized from Schumpeter's perspective since the innovative vision of other sectors of the economy, including transportation, needs to be constructed.

Also, the microeconomic consequences of the COVID-19 pandemic have been the subject of recent studies. Cullen (2020) analyzed the impact of the pandemic on food supply chains. Stiller and Zink (2020) showed the situation in the European banks during the pandemic. Korzeb and Niedziółka (2020) described the resistance of commercial banks in Poland to the crisis caused by the COVID-19 pandemic. Zhang et al. (2020) show the pandemic's impact on transport services in China, taking into account the virus's spatial dissemination. Gallego and Font (2020) investigated changes in the air passenger demand due to the COVID-19 crisis. They analyzed implications for tourism policies.

An interesting study on the effects of the COVID-19 pandemic on business and research was presented by Donthu and Gustafsson (2020). They reported the recent literature review on that issue in the following perspectives: consumer behavior, markets, and predicted lasting effect. Negative consequences of the pandemic in small and medium businesses in the USA are the subject of the research revealed by Senz (2020). She found out that 43% of businesses have been temporarily closed, and 40% of the labor force has been laid off or furloughed since late January. Shafi et al. (2020) analyzed the impact of the pandemic crisis of micro, small, and medium industry in Pakistan in the context of business survival. They surveyed 184 enterprises and found that 2/3 may not survive until the end of the year. A simulation study concerning the pandemic's consequences for the supply chains (SC) and policy recommendations are provided by Ivanov (2020). He found out that the timing of the closing and opening of the facilities at different echelons might become a significant factor determining the epidemic outbreak's impact on the SC performance. Other essential factors are lead-time, speed of epidemic propagation, and the upstream and downstream disruption durations in the SC. Amankwah-Amoah (2020) indicated how the aircraft companies have adjusted to the COVID-19 outbreak essential for the road transport industry. Tardivo et al. (2021) showed the impact of the COVID-19 pandemic for various transport modes. Świtała and Łukasiewicz (2021) examined 100 enterprises representing the road transport sector. They found out a destructive impact of the pandemic on the activities carried out in road transport. Consequences of the pandemic can be seen both in the reduction of transport services' demand and the poorer financial condition of the companies surveyed. The authors showed that transport companies applied a cost-cutting policy.

The study's purpose is to examine the vulnerability and resilience of road transport enterprises in Poland—especially in international transport—for the economic crisis that resulted from the first phase of the pandemic. The first phase is essential for the analysis because it allows analyzing how much the road transport sector was insensitive to the crisis. The first phase differs from the sequent ones since it could not be predicted in advance, so intense but short-term shock results are most valuable. The study was projected to identify the main challenges of the road transport sector at the beginning of the pandemic and provide a deep insight into the managers' activity to use the opportunities provided by the market and institutional environment.

The term vulnerability is adopted from macroeconomic literature. Typically it is defined by a set of indicators. The International Monetary Fund (IMF) defines vulnerability in four perspectives, i.e., the government, the financial sector, and the household and corporate sectors. The corporate sector focuses on the potential impact of exchange rate and interest rate changes on corporate sector balance sheets. Indicators related to corporate leverage, profitability, cash flow, and financial structure are also relevant (https://www.imf.org). On the other hand, in logistics literature, vulnerability is defined as a susceptibility to change or loss because of existing organizational or functional practices or conditions (Barnes and Oloruntoba 2005). Both perspectives are relevant to this study.

This paper contributes to the existing literature in several ways. Firstly, it attempts to investigate the lockdown's economic and financial effects caused by the COVID pandemic in the road transport industry. We collected primary data using a specially designed questionnaire addressed to entrepreneurs managing road freight transport enterprises. This group of entrepreneurs' choice was dictated by the fact that Polish haulers transport the most goods from all European countries, competing for primacy with German entrepreneurs. 500 randomly selected enterprises were examined in the study among about 4,500 registered enterprises.^1^ Secondly, we identify essential factors for road transport enterprises' economic performance imposed on the pandemic crisis. The current study applies an ordered logit model, which is well-fitted for individual data analysis and allows separating enterprises according to the vulnerability level to discover these factors. Thirdly, the results of the survey seem to support the creative destruction concept.

The remainder of the work is structured as follows. The next section analyses the pandemic's road transport sector, while the third section characterizes the data collection process and the methodology, i.e. the econometric ordered logit models. In the fourth part, the description of the results of the survey is presented. In ``Vulnerability and resilience of transport enterprises–the results from empirical ordered logit models'' section, the estimated models are presented along with the interpretation. Discussion is provided in ``Discussion'' section. The last part presents conclusions and recommendations resulting from the research conducted.

## International road transport of goods under the conditions of the COVID-19 pandemic

The COVID-19 pandemic started in December 2019 in a Chinese province—Hubei, and spread worldwide, including Europe, in March 2020. To protect citizens' health, many countries introduced different limitations, including an ordered lockdown. In Europe, many countries experienced the first lockdown cases between 11 and 24 March 2020 (Kufel 2020). The consequences of the COVID-19 pandemic, primarily related to individual and social health, healthcare systems, touched other social life fields like family life, education, culture, tourism, a system of work, and entire economies. Moreover, the pandemic is still very strong and expanding in some areas more heavily than others. The forecasts for the next 12 months show how the pandemic is unfolding (Kufel 2020). An increasing number of real cases reported by the John Hopkins University (Dong et al. 2020) confirms this tendency, despite starting vaccinations at the beginning of 2021. The peaks of new cases were registered in December 2020-January 2021, April 2021, and the latest growing in August 2021.^2^

**Fig. 1:**
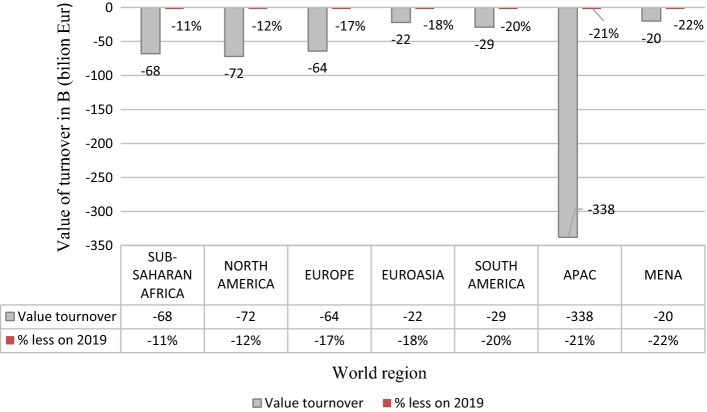
Turnover impact by region road freight transport – forecast for 2020. *Asia and Pacific region (APAC),* the Middle East and North Africa (MENA). *Source*: (IRU 2020b)

The transport industry was one of the branches that suffered much from the pandemic, particularly at its early stage in 2020. According to the report prepared by the International Road Transport Union (IRU), goods transport losses globally were expected to exceed EUR 550 billion, with annual turnover down 18%, and passenger transport losses in Europe are expected to exceed EUR 80 billion, with annual revenue down 57% (IRU 2020a). Furthermore, the regional predictions were very pessimistic. Figure 1 presents the estimated losses in road freight transport in a geographical breakdown. The most significant losses are expected in the Asia–Pacific area.

The IRU Annual Report 2020, issued in 2021 (IRU 2021a), revealed that revenue losses for the global road transport sector alone, both passenger and goods, were almost one trillion USD. Further data concerning the performance of road transport of goods show that the loss in revenues in 2020 gained 679 billion USD and the insolvency measures were critical. On the other hand, global losses for the goods road transport sector are expected to reach 347 billion USD in 2021 (IRU 2021b). The numbers indicate that in the second, third, and following pandemic waves, the road transport industry could manage the unpredicted shock in the first quarter of 2020. However, the effects of the crisis will last long.

When considering the profile of transportation on the road in Poland, it should be noted that until the end of 2019, Poland was the leader in this industry in Europe, with the highest market share of 17.58%. In the years 2012–2019 Polish transport sector experienced the dynamics of + 5.8% yearly on average, which was the highest value in comparison to Germany (+ 0.2%), Spain (+ 2.86%), France (+ 0.51%), Italy (+ 1.34%) and United Kingdom (+ 0.78%). In Poland, international cargo transport accounts for over 60% of the entire transport volume share, while in 2019, it was 65.79%. At the end of 2019, Polish carriers had over 246 thousand trucks to provide international transport services. According to the PwC report from 2019 (PwC Report 2019), Polish transport had the opportunity to strengthen its competitiveness, thanks to the German market's proximity and the reliability of its services and affordable price offers.

Innovation such as implementing the information technology (IT) solutions in Polish transport enterprises increased transport service quality. During the pandemic, transport performance was exposed to many restrictions and a significant reduction in production and trade, which resulted in a decreased number of transport orders. According to Timocom (2020), one of the German most extensive transport exchanges, due to a significant reduction or even stoppage of production processes in some branches, i.e., automotive, the number of orders for Germany's services decreased by about 15%.

The COVID-19 pandemic consequences threaten not the entire transport industry. One should notice that the growing number and value of online buying orders during the lockdown caused the courier posts market to be one of the best developing during the pandemic. As was shown in the report E-commerce in Poland (2020) prepared by Gemius, Poland, 73% of internet users buy online. When compared with 2019, this result is higher by as much as 11 p. It was also confirmed by the Chamber of Electronic Economic report, which indicates that over 53% of the Internet users surveyed made online purchases (EIZBA 2020). The INELO report, which resulted from a survey conducted in April 2020 among 800 road transport enterprises (Infowire 2020), shows that the smallest decrease in the number of kilometers can be seen on Poland's routes. The reports indicate that domestic demand could mitigate the fall in international markets.

It is obvious that in some branches the demand for transport services increased. Ivanov and Desgui (2020) showed that demand has drastically increased for some supply chains (e.g., facial masks, hand sanitizer, disinfection spray). The supply was not able to cope with that situation. For others (e.g., automotive industry), the demand and supply have drastically dropped, resulting in the production stops, causing the danger of bankruptcies and necessities of governmental supports. This fact motivated the authors to consider the viability of a supply network, defined as “a system able to meet the demands of surviving in a changing environment”. Viability is an opposite concept to resilience. The authors elaborated conceptualization of a novel decision-making environment that considers supply networks and viability as integrity to ensure survivability at a large scale. They argue that the recent coronavirus COVID-19 outbreak showed that in the case of extraordinary events, supply chain resistance to disruptions needs to be considered at the scale of survivability or viability to avoid supply chain and market collapses secure the provision with goods and services. Loske (2020) analyzed the impacts of panic buying and increasing home consumption on transport volume and freight capacity dynamics in German food retail logistics in the narrower range. The results of the study showed that the increasing freight volume for dry products in retail logistics did not depend on the duration of the COVID-19 pandemic but the strength quantified through the total number of new infections per day. These findings enabled us to derive detailed conclusions on transport units capacities and short and long terms contracts.

We can state that several qualitative changes in road transportation are already observed. They correspond to the Schumpeterian perspective which allows treating the pandemic as a discontinuity (a structural break) (Foster and Kaplan, 2001). One of them is the electronic freight transport information (eFTI) regulation that has been approved by the EU and will enter into force in August 2024. It establishes a legal framework for transport operators to share information with enforcement authorities in an electronic format. At the moment, multiple IT solutions are being used throughout Europe to exchange freight transport information. The incompatibility of these solutions often leads drivers to prefer paper documents, a costly and time-consuming process. As a uniform legal framework, eFTI will standardize the use of electronic information, which will encourage transport operators to shift towards digital solutions. It will also facilitate enforcement work, reducing waiting times during inspections (IRU 2021a; www.iru.org).

Other innovative activities such as increased online trade and the necessity of 'door-to-door' delivery for both business to business (B2B) and business-to-customer (B2C) operations. Two other positive results of the COVID-19 pandemic are mentioned in the International Road Transport Union (IRU 2020a). These are exemptions on driving and resting times rules and the extension of driving licenses and certificates (Regulation 2020). Also, a temporary reduction in daily global CO_2_, NO_2,_ and other emissions due to limited demand for energy, including transportation, can be considered favorable for sustainable development (Aloi et al. 2020; Le Quéré et al. 2020; Sharma et al. 2020). The COVID-19 pandemic, together with a changing legal environment caused by the EU mobility package (www.iru.org), is still a source of a big challenge for the transport industry in Poland.

## Data and methodology

### Sample characteristics

The survey aimed to determine factors of vulnerability and resilience for the pandemic crisis's consequences in June-July, 2020, among 500 randomly selected road transport enterprises that carry goods using the CATI method. To obtain correct and complete results of the questionnaire survey, the population of respondents was defined. The surveyed units were defined in terms of both material criterion, *i.e*., enterprises providing road transport, and territorial criterion, *i.e*., enterprises whose business activity is registered in Poland. The survey was carried out based on a sample of enterprises from the databases of members of the Association of International Road Carriers in Poland. As a result, the population from which the enterprises were drawn was 4,500 enterprises. The sample size equals 500 because if one company denied answering the questions, it was replaced by another one from the list. Finally, it gave 11.11% of the entire population. An essential element for the study is that decision-makers took part in the questionnaire, *i.e.,* managers with appropriate competencies in the scope of the answers provided. The statistical assumption dictated the number of 500 respondents that the maximum error rate in the estimation would not exceed 5% (Aczel and Sounderpandian 2002). Table 1 presents the structure of the sample.

**Table 1:** Structure of the studied sample according to selected criteria

*Participation of enterprises by the form of conducted activity*	*%*	*The share of enterprises by the number of employees*	*%*
Sole proprietorship	2.20	From 1 up to 9	6.60
Limited liability company	80.80	From 10 up to 49	56.00
General partnership	13.80	From 50 up to 249	32.40
Joint-stock company	2.20	Over 250 employees	5.00
Other (limited partnerships, other)	1.40		
*Participation of enterprises by the homogeneity of the conducted business activity*	*%*	*The share of enterprises by the number of employed drivers*	*%*
At least 80% of revenues generated from transport activities	74.20	From 1 up to 9	20.00
Less than 80% of revenues generated from transport activities*	25.80	From 10 up to 49	53.00
		From 50 up to 249	24.00
*The type of transport*	*%*	Over 250 employed drivers	3.00
Universal	93.20	*The share of enterprises by the number of transport units*	%
Special (cold stores, cisterns)	6.80	From 1 up to 4	3.6
*Participation of enterprises by experience in running the business activity*	%	From 5 up to 10	19.8
More than 10 years	95.00	From 11 up to 20	22.0
From 5 up to 10 years	2.80	From 21 up to 50	28.2
From 3 up to 5 years	1.20	From 51 up to 100	13.2
Up to 3 years	1.00	From 101 up to 200	7.4
		Above 200	5.8
*The range of services provided*	*%*	*The leading direction of the service portfolio*	*%*
International	66.20	Poland—Other EU countries	57.80
Domestic	23.40	Country	33.00
Regional	7.10	Poland—East of Europe	4.80
Local	3.30	Inside the EU—outside Poland	4.40

Analyzing the data presented in Table 1, one can conclude that the sample of 500 enterprises selected for the study roughly reflects the structure of transport enterprises providing domestic and international transport services, being in line with the report (GITD 2020). Furthermore, the research sample analysis indicated that it was a homogeneous activity for 371 enterprises (74.2%) in which over 80% of revenues were generated from road transport. In 25.8% of cases, the activity was not homogeneous, which meant that income sources were diversified. However, for only 14.8% of enterprises, revenues from transport activities accounted for less than 50% of their total revenues. 92.3% of enterprises offered universal transport activity, and only 6.8% of enterprises performed specialized transports. In 89.6% of cases, companies carried out tasks related to both international and domestic transport, and the leading direction (57.8%) was the European Union countries. 86.8% of companies had a license for domestic and international transport, and only 15% of transports were carried out to meet the 'own needs.' In the selected sample, 95% of enterprises have been operating in the transport services market for over ten years, which means that they have achieved a strong position in the transport market and meet the existing conditions for transport services under the pandemic. Analysis of the survey data reveals that all enterprises participating in the survey had vehicles with telematics systems installed. In total, all surveyed enterprises owned 24,975 vehicles, 97.3% of which were equipped with a telematics system (24,304 vehicles). This is in line with the possibility of participation in multimodal transport and the supply chain management rules (see, Asborno et al. 2021).

### Questionnaire construction

The questionnaire was constructed based on the microeconomic knowledge, literature review (Osińska and Zalewski 2012; Vochozka et al. 2016), and recent legal acts introduced in the period of the COVID-19 pandemic (Anti-Crisis Shield 1.0 – 4.0; see: Anti-Crisis Shield KPMG services 2020). The complete list of questions used in the study is presented in Table 2. Symbols Y and X correspond to the variables used in the ordered logit model described in sections ``Ordered logit models—an overview'' (theory) and ``Vulnerability and resilience of transport enterprises–the results from empirical ordered logit models'' (empirical results).

**Table 2:** Questions and variables definition

Variable name	Question	Scale of measurement
X1	Has the announcement of the state of the epidemic in Poland as well as the pandemic on a global scale had a significant impact on reducing the number and scope of transport in the enterprise?	Nominal
X2	Use a percentage scale (0%—100%) to describe the decrease in the number of orders processed in this period	Metric
X3	Use a percentage scale (0%—100%) to describe the increase in the number of orders processed in this period	Metric
Y1	Indicate on a scale from 1 to 7 to what extent the closure of the borders affected the decrease in the number and value of shipments (1- we were not affected by the change, 7- severely affected)	Ordinal (Likert)
X5, X6	Has the enterprise reduced the employment of drivers as a result of a reduced volume of transport? If yes, what is the reduction expressed as a percentage?	Nominal, metric
X7, X8	Have you used the deferred payment of leasing instalments? If yes, how many months?	Nominal, metric
X9	Did the increase in the EUR/PLN exchange rate allow you to compensate for the losses resulting from the decrease in transport?	Nominal
X10	Did the decrease in fuel prices allow you to compensate for the losses resulting from the decrease in transport?	Nominal
X11	Has the company's profitability decreased due to the COVID-19 pandemic?	Nominal
X12	By how much did the company's profitability decline during the pandemic (0%; less than 1%; 2–3%; 4–5%; more than 5%; Our company’s profitability increased despite the pandemic)	Metric
X13	Has the pandemic situation forced a change of existing contractors and industries served by the enterprise?	Nominal
X14	Have you used the support of the government's anti-crisis shield programme?	Nominal
X15	Rate on a scale from 1 to 7 to what extent the solutions of the anti-crisis shield supported the functioning of the enterprise (1- low support, 7- strong support)	Ordinal (Likert)
X16	Have the transport associations provided the enterprise with organizational support during this period?	Nominal
X17	Does the persistence of the COVID-19 pandemic pose a long-term threat to the company's transportation operations?	Nominal
X18	Does the pandemic make the transport enterprise exposed to the risk of bankruptcy?	Nominal
X28	How long has the company been operating on the road transport market (in years)?	Metric
X29	What is the number of transport units used to carrying goods in the enterprise?	Metric
X30	What is the number of transport units in the enterprise that are equipped with telematics devices?	Metric
X32	What is the total number of employees in the enterprise, regardless of the form of employment?	Metric
X33	What is the number of drivers in the enterprise, regardless of the form of employment?	Metric

It is worth noting that the variables Y1 and X15 were ordered logically on a seven-point Likert scale, where 1 means ‘no impact’ and 7 represents ‘a severe impact’. The Likert scale is typically defined as a five (or more) point scale, which allows the individual to express how much they agree or disagree with a particular statement. It falls within the ordinal level of measurement (Likert 1932; Jamieson 2004; McLeod 2019). Other variables were measured on nominal and metric scales.

### Ordered logit models—an overview

In the current study microunits i.e., road transport enterprises were examined for being vulnerable or resilient to the COVID-19 pandemic negative effects. The econometric logit model was selected as a tool of quantitative analysis. The limited endogenous variable models, such as a logit model, are intended to predict the probability of satisfying conditions defined by the researcher. In the ordered form, the endogenous variable is defined as follows:

1 $$y_{i} = \left\{ {\begin{array}{*{20}c} 1 & {\text{if variant 1was selected}} \\ \cdots & \cdots \\ k & {\text{if variant k was selected}} \\ \end{array} } \right.$$

where $i = 1,2, \ldots ..,n$ denotes sequent observations corresponding to the questionnaire respondents, where n is a sample size. Variants $1, \ldots .,k$ correspond to the variants of the answer. It often occurs in the case of modeling a sequentially ordered set of variants.

As the endogenous variable has only a few variants and exogenous variables can be either metric or non-metric, it is necessary to transform the model to obtain consistent estimates. The dependent variable and its transformation are then defined as follows. Firstly, let us assume that the ordered variable $y_{i}$ (observed) represents a particular case of a continuous (latent) variable

2 $$y_{i}^{*} = \beta_{0} + \mathop \sum \limits_{k = 1}^{K} \beta_{k} x_{ki} + e_{i}$$

where $y_{i}^{*}$ is a transformed endogenous (latent) variable; $x_{ki}$ is a $k^{th}$ observed exogenous variable, $\beta_{0}$ is a constant, $\beta_{k}$ is a model parameter, $e_{i}$ is an error term and $i = 1,2, \ldots ..,n$ denotes sequent observations. A variable $y$ is observed and classified according to several $J$ values (variants), which correspond to natural numbers. Mapping $y^{*}$ on $y$ is monotonic. Firstly we define $J + 1$ cut points, namely $\alpha_{j + 1}$, $j = 1,2, \ldots .,J$, which divide the entire domain of $y^{*}$ into several intervals such that $y = j$ if $y^{*}$ belongs to the interval limited by $\alpha_{j - 1}$ and $\alpha_{j}$. It is assumed that $\alpha_{0} = - \infty$ and $\alpha_{J} = + \infty$.

The mapping is then defined as follows:

3 $$\begin{array}{*{20}c} {y_{1} = 1} & \leftrightarrow & { - \infty < y_{i}^{*} \le \alpha_{1} } \\ {y_{1} = 2} & \leftrightarrow & {\alpha_{1} < y_{i}^{*} \le \alpha_{2} } \\ {and \ so \ on} & \cdots & \cdots \\ \end{array} .$$

Replacing (3) with the RHS of the model (2) without a constant term and re-arranging, we obtain the ordered logit model:

4 $$\begin{array}{*{20}c} {\alpha_{j - 1} < y_{i}^{*} \le \alpha_{j} } \\ {\alpha_{j - 1} < \mathop \sum \limits_{k = 1}^{K} \beta_{k} \le \alpha_{j} } \\ {\alpha_{j - 1} - \mathop \sum \limits_{k = 1}^{K} \beta_{k} x_{ki} < e_{i} \le \alpha_{j} - \mathop \sum \limits_{k = 1}^{K} \beta_{k} x_{ki} } \\ \end{array}$$

which corresponds to

5 $$\begin{aligned} p_{ij} = &\, P\left( {y_{i} = j} \right) = P\left( {\alpha_{j - 1} - \mathop \sum \limits_{k = 1}^{K} \beta_{k} x_{ki} < e_{i} \le \alpha_{j} - \mathop \sum \limits_{k = 1}^{K} \beta_{k} x_{ki} } \right) \\ = &\, {\text{P}}\left( {e_{i} \le \alpha_{j} - \mathop \sum \limits_{k = 1}^{K} \beta_{k} x_{ki} } \right) - P\left( {e_{i} < \alpha_{j - 1} - \mathop \sum \limits_{k = 1}^{K} \beta_{k} x_{ki} } \right) \\ = & \,F\left( {\alpha_{j} - \mathop \sum \limits_{k = 1}^{K} \beta_{k} x_{ki} } \right) - F\left( {\alpha_{j - 1} - \mathop \sum \limits_{k = 1}^{K} \beta_{k} x_{ki} } \right) \\ \end{aligned}$$

To estimate parameters $\beta_{k}$ and $\alpha_{j}$ simultaneously and to identify the parameters, a constant term must be excluded from the specification. Two issues concerning model (4) must be mentioned. The first is related to an estimation that is effectively ensured using the Maximum Likelihood method. The second one refers to the proportionality of odds in the model (4). It can be shown that the odds ratio in the model (4) is constant, which means that the parameters $\beta_{k}$ are estimated at the same level across all variants of $y_{j}$ (Gruszczyński 2010, p. 121). Such an assumption can be too strong in some cases. Then the generalized ordered logit model (GOM) is suggested.

The generalized ordered model assumes that the threshold ($\alpha_{j} )$ values are linear functions of explanatory variables, i.e. $\alpha_{j} = \tilde{\alpha }_{j} + \mathop \sum \nolimits_{k = 1}^{K} \gamma_{k} x_{ki}$ and a constant term is allowed. The formula (5) changes into:

6 $$p_{ij} = F\left( {\tilde{\alpha }_{j} - \mathop \sum \limits_{k = 1}^{K} \tilde{\beta }_{j} x_{ki} } \right) - F\left( {\tilde{\alpha }_{j - 1} - \mathop \sum \limits_{k = 1}^{K} \tilde{\beta }_{j} x_{ki} } \right)$$

where $\tilde{\alpha }_{j}$, $\gamma_{k}$ and $\tilde{\beta }_{j}$ are estimated jointly using the Maximum Likelihood method.

It should be noted that the assumption of proportional odds is eliminated in (6), but the GOM remains linear. It means that model parameters differ across the threshold values $\tilde{\alpha }_{j}$ corresponding to the variants of the endogenous variable *y*_*i*_*.*

A sequence of indicators measures the quality of the logit model. They are widely described in Cameron and Trivedi (2008) and Gruszczyński (2010). One of the simplest indicators is pseudoR2, defined by McFadden (1974). It should be noted that in logit models, this indicator is typically on a low level. Testing for individual parameter significance uses standard *z* statistics, and the joint significance of the model is based on the likelihood ratio (LR) chi-squared distributed test. In the null hypothesis, parameter’s insignificance is assumed, so rejecting the null supports the existing relationship and further inference.

The current study applied Wolfe and Gould's test for proportional odds to decide whether the generalized ordered model should be estimated (Wolfe and Gould 1998). In the null, proportional odds are assumed. The test statistic is chi-squared distributed. If the data rejected the null, the general ordered model was estimated. There is yet another possibility. Some of the proportionality assumptions are satisfied, and some are not. Then a partial proportional odds model, being a combination of (5) and (6), is estimated (Williams 2006).

## The road transport industry in Poland under the pandemic restrictions

Based on the sample and a questionnaire characterized in ``Data and methodology'' section, we started with a descriptive analysis of the answers provided by the transport managers.

A huge share of the road transport enterprises in Poland (95.8%) confirmed that the announced state of the pandemic in March 2020 had a significant impact on reducing the number of transport operations. In detail, the structure of decrease was as follows: 63.6% of the respondents indicated that reduction in the number of orders in the period March – July 2020 amounted to 20%, 31.8% indicated the interval between 20 and 35%, and 4.4% enterprises indicated that the decrease in the demand amounted to 40% and more, including five enterprises which indicated that the decrease was 80%.

The temporary closure of Poland's exit borders and the resumption of controls at transit borders also significantly affected international transport operations. The respondents' answers show that 88.4% indicated a range from 5 to 7 on the seven-point Likert scale. In comparison, 42.6% considered the changes highly significant for reducing the number and value of transports (the value of 7 on the Likert scale).

It is worth emphasizing that employment was practically not reduced during the first phase of the pandemic. Only 6.2% of the respondents reduced the number of drivers employed, and 3% reduced employment by a maximum of 6% of their resources.

Only 19% of the respondents took advantage of the possibility of postponing leasing installments, which was a small ratio and may suggest that the financial situation of enterprises has been stable at the beginning of the COVID-19 period.

An essential factor influencing profitability while maintaining a constant freight level in executed orders is the euro/PLN exchange rate in international transport. The analysis of the respondents' answers shows that only 8.4% of the answers were positive. 91.6% of the respondents replied that the depreciation of PLN vs. euro did not compensate for the reduced number of transport orders processed.

The respondents were asked whether the low unit cost of fuel (PLN net per 1 L) could maintain or even increase its profitability. Only 15.8% of the responses indicated that the low fuel price made up for the losses incurred. However, 84.2% of the respondents indicated that the fuel price did not compensate for the losses incurred.

Due to the maintenance of the current position in the transport market and enterprises' financial stability, the decline in profitability was important; 90.8% of the respondents indicated that their profitability had decreased due to the pandemic, 1.8% indicated that it was maintained at the current level, and 7.4% could not answer this question unequivocally. The reduction structure was as follows: 60% of respondents indicated that its scale exceeded 5%, 14.2% indicated a decrease of 4, and 17% replied that it was between 2 and 3%. 8.4% of the respondents indicated that this decrease was less than 1% or was 0%.

In the survey, the respondents were asked whether the situation forced a change of existing contractors and industries served. As a result, 70.4% of respondents indicated that the pandemic situation did not force them to seek new contractors because their volume had significantly decreased, and 16.4% of respondents indicated the need to look for new contacts to solve the existing situation. In 13.2% of cases, no precise answer to the question was given.

An essential element that probably allowed road transport entrepreneurs to survive was the Polish government's decision, which, in the form of legal acts, provided support in various fields related to running a business, but above all relating to tax and direct financial support. Among the examined enterprises, 98.5% took advantage of the proposed various forms of support. As many as 77.6% of those benefiting from the government support indicated five or more on the Likert scale, and 39% assessed the scope and form of aid are essential (value seven on the scale). In turn, 84.8% benefited from the assistance of the Social Insurance Institution, *i.e*., from the exemption from paying compulsory social insurance contributions for three months, and 69.6% from the possibility of financial support provided by Labour Offices in the form of payment of a part of remuneration to employees. Non-returnable loans, which were used by 31% of respondents, and loans granted by the Polish Development Fund (PDF), also turned out to be significant support for enterprises. 35.6% of them benefited from this form of support. The support from the PDF could be obtained only in the case of a significant (25%) decrease in the company's month-on-month or year-on-year turnover for selected months. As a rule, 75% of loans granted from the PDF will be written off to entrepreneurs.

The most critical element of each company is financial liquidity, which, due to the forecasts about the development of the situation, caused some payment gridlocks. When asked about the most crucial bankruptcy risk factors under a long-term pandemic situation, 83.4% of answers indicated financial liquidity loss. As the second (67.4%), they mentioned the fear of losing their recipients.

For 74.6% of the respondents surveyed, the persistence of a pandemic over longer than 6–8 months and the failure to open up the economy will be a significant cause of bankruptcy. When the survey was being carried out, more than half of entrepreneurs (53.8) feel threatened, while 46.2% believe that there are no grounds for fear related to possible bankruptcy.

Unfortunately, nearly 90% of the respondents examined state that they lack support from the transport associations they belong to and that substantive support would be essential, for example, in the preparation of documentation related to the anti-crisis shield, support in contacts with customers on the international market, or, finally, launching support with institutions controlling road transport.

One question focused on innovative solutions mainly based on the IT systems and data analysis in a remote form. The current study revealed that due to the COVID-19 pandemic, the enterprises extensively used GPS systems and Transport Management Systems to determine drivers' work, ensure workflow, and guarantee employees' security. It seems that there was a permanent and irreversible change in the operating management area, which is a positive element of the situation. This fact is in line with the creative destruction assumptions since it shows transport companies' positive and innovative attitude towards the challenges caused by the pandemic crisis. It also confirms that Polish enterprises are ready for the electronic freight transport information (eFTI) regulation, as proposed by the European Union. There is another advantage of digitalization of transport documents, namely it allows efficient management of both transport processes and the enterprise. This is an actual innovation in transport companies, which used IT technology to track transport units, but they did not used the collected information for operational management (Zalewski 2019).

As follows directly from the responses, road transport enterprises in Poland were vulnerable to the crisis caused by the COVID-19 pandemic. The anti-crisis policy tools weakened enterprises' exposure to this crisis in the short run. Still, they were of an *ex-post* nature being aimed at mitigating the labor market's effects and reducing the current financial burden. Market issues such as acquiring new orders had to be handled by the companies. The global decline in fuel prices in the world markets accompanying the pandemic additionally caused a decrease in the operating costs of transport companies, which, in turn, increased their resistance to the effects of the crisis.

It is worth mentioning that the paper investigates primarily data collected in 2020 directly from managers of the transport enterprises. Thus many of their opinions could be though subjective. Therefore, various activities were undertaken to minimize the responses subjectivity. Firstly, the reports on the situation in the road transport sector provided by the International Road Transport Union and the International Labor Organization in 2019 and 2020 were analyzed. Secondly, a sample transport company was observed concerning its economic and financial performance as an anonymous example. Thirdly, we provided a pilot study on 30 enterprises before starting the reported study. Based on the information gathered from the above sources, we confirmed that the presented results are trustworthy and valid.

## Vulnerability and resilience of transport enterprises–the results from empirical ordered logit models

In this section, the empirical ordered logit models are presented to evaluate the vulnerability and resilience of the enterprises operating in the road transport sector. As the sample of enterprises is both randomly selected and representative, the generalization of the results is allowed. Furthermore, the sample size ensures statistical inference at a 5% significance level.

Three models were estimated in the following subsamples: a total sample of 500 enterprises, medium enterprises only (189 units), and micro and small enterprises only (311 units). It enables to distinguish the factors of enterprises’ sensitivity and provide recommendations directed not only to the entire sector but also to the sub-groups. The endogenous variable Y1 was a multinomial ordered variable observed on the seven-point Likert scale (see Table 2). It corresponds to the scale that after closing the borders due to the COVID-19 pandemic threat, the number of orders and value of transporting goods was smaller. The answer '1' denotes no change, and the answer '7' means that the change was severe. Due to the low number of answers indicating 1, 2, and 3, the variable was transformed according to both the scale and answers. In the models the following cases appear: 1 = 'a small decrease', 2 = 'a lower medium decrease', 3 = 'an upper-medium decrease', 4 = 'a high level of decrease'. The answers' distribution was as follows: 1- selected by 58 respondents, 2–by 112, 3 by 117, and 4 by 213 respondents. It is clear that 42.6% selected a high decrease in demand for their services.

All exogenous variables used in models are defined in section ``Questionnaire construction'' (Table 2). At the initial stage, all variables were included in the model. Insignificant variables were eliminated from the estimated models using stepwise regression (Harrell 2001). The presented results were calculated using the STATA14 software. In the study, models (5) and (6) were estimated sequentially. Wolfe and Gould's test for proportional odds was applied to decide which one is apprppriate (Wolfe and Gould 1998). As for each subsample model (5) was rejected as not satisfying the proportional odds condition, the partial proportional odds models being a combination of models (5) and (6) are presented in Table 3. The proportional odds test results and other model diagnostics presented in Table 3 refer to the partial proportional odds models.

**Table 3:** Parameters estimates of general ordered logit models for factors of decrease in number and value of shipments (Y1)

Variable		X2	X8	X10	X11	X12	X15	X18	× 29	× 32	Constant
Model 1. Total *n* = 500											
alpha1 = 3.059 [0.596]											
Coeff		**0.103**		**−0.833**	**1.541**	**0.598**	**0.612**	**−1.095**			**−2.819**
Std error		**0.025**		**0.276**	**0.420**	**0.170**	**0.253**	**0.517**			**0.574**
z-stat		4.12*		−3.02*	3.67*	3.52*	2.42*	−2.12*			−4.91*
alpha2 = 5.271 [0.640]											
Coeff		**0.025**		**−0.833**	**1.541**	**0.598**	**0.387**	0.622			**−3.649**
Std error		**0.013**		**0.276**	**0.420**	**0.170**	**0.149**	0.355			**0.543**
z-stat		1.92*		−3.02*	3.67*	3.52*	2.60*	1.75			−6.72*
alpha3 = 7.200 [0.673]											
Coeff		**0.056**		**−0.833**	**1.541**	**0.598**	**1.123**	**2.671**			**−9.566**
Std error		**0.013**		**0.276**	**0.420**	**0.170**	**0.184**	**0.486**			**0.869**
z-stat		4.31*		−3.02*	3.67*	3.52*	6.10*	5.49*			−11.01*
Model Diagnostics:	Log-likelihood	−440.90	Pseudo R2		0.316		Proportional odds test		Chi2[14] = 135.63 [0.000]		
Model 2. Medium *n* = 189											
alpha1 = 1.871 [0.624]											
Coeff		**0.693**	**0.669**	**−1.648**		**0.768**	0.332	**−4.388**			**−8.04**
Std error		**0.183**	**0.219**	**0.591**		**0.316**	0.273	**1.942**			**1.925**
z-stat		3.78*	3.06*	−2.79*		2.43*	1.22	−2.26*			4.18*
alpha2 = 4.007 [0.718]											
Coeff		0.009	−0.044	**−1.648**		**0.768**	0.332	**2.364**			**−1.908**
Std error		0.025	0.123	**0.591**		**0.316**	0.273	**0.965**			**0.797**
z-stat		0.38	−0.36	−2.79*		2.43*	1.22	2.45*			−2.39*
alpha3 = 6.597 [0.880]											
Coeff		**0.057**	0.068	**−1.648**		**0.768**	0.332	**3.432**			**−6.125**
Std error		**0.026**	0.241	**0.591**		**0.316**	0.273	**0.973**			**1.306**
z-stat		2.26*	0.28	−2.79*		2.43*	1.22	3.53*			−4.69*
Model Diagnostics:	Log-likelihood	−107.13	Pseudo R2		0.546		Proportional odds test		Chi2[6] = 8.00 [0.238]		
Model 3. Micro and small *n* = 311											
alpha1 = 5.080 [1.478]											
Coeff		**0.066**		**−1.369**	**1.652**	**0.436**	0.544	−0.716	**0.128**	**−0.068**	**−2.138**
Std error		**0.029**		**0.5113**	**0.579**	**0.217**	0.351	0.654	**0.036**	**0.021**	**0.981**
z-stat		2.28*		−2.68*	2.85*	2.01*	1.55	−1.09	3.48*	−3.30*	−2.18*
alpha2 = 7.387 [1.507]											
Coeff		0.021		−0.106	**1.652**	**0.436**	0.134	0.598	**0.071**	**−0.068**	**−2.563**
Std error		0.019		0.369	**0.579**	**0.217**	0.204	0.426	**0.027**	**0.02**	**0.855**
z-stat		1.10		−0.29	2.85*	2.01*	0.66	1.40	2.57*	−3.30*	−2.99*
alpha3 = 9.236 [1.538]											
Coeff		**0.113**		**−3.287**	**1.652**	**0.436**	**1.595**	**3.079**	**0.116**	**−0.068**	**−12.784**
Std error		**0.027**		**1.360**	**0.579**	**0.217**	**0.358**	**0.855**	**0.030**	**0.020**	**1.711**
z-stat		4.17*		−2.42*	2.85*	2.01*	4.46*	3.60*	3.86*	−3.30*	−7.47*
Model Diagnostics:	Log likelihood	−239.286	Pseudo R2		0.408		Proportional odds test		Chi2[6] = 7.73 [0.258]		

^*^Denotes z-statistic significant at 0.05 significance level, additionally significant values are bolded

In Table 3, the estimated multinomial ordered logit models for Y1 have been shown. The table consists of three parts, including a model for a total number of enterprises (Model 1), a medium enterprise model (Model 2), and a micro and small enterprises model (Model 3). In each part, except parameter estimates, their standard errors, and z-statistic for the parameter’s significance, the parameter estimates corresponding to thresholds' (alphas) under which the endogenous variable is observed in the sample are presented. In each part, model diagnostics are provided. The estimation method was the maximum likelihood, which enables joint estimation of all parameters. All variables were tested for proportionality across the variants indicated by the parameters alpha. The number of alphas is 3 as shown in the formula (3). If the proportionality condition was satisfied, the parameter estimates remain unchanged across the variants indicated by alphas. Thus, the resulted model is a partial proportional odds model (Williams 2006, 2016).

A profound econometric inference was based on all answers presented by the respondents. To check whether parameter estimates are robust, the bootstrap standard errors were estimated (available for request). The obtained results satisfy all statistical requirements. Additionally, positive signs of the parameters estimates indicate vulnerability, while negative signs correspond to enterprises' resilience for the decrease in number and value of transport.

The results showed the following factors of enterprise vulnerability for the demand shock caused by the pandemic: a decrease in the number of orders (X2), a decrease in profitability (X11), a scale of decrease in profitability (X12), and the government anti-crisis policy implementation (X15). This picture refers to all analysis samples (total, medium, micro, and small enterprises). The main factor of resilience for the shock caused by the pandemic is represented by the decrease in truck fuel prices (X10). The parameters estimates are negative across all subsamples and all variants of analysis. The case of X18, representing the threaten of bankruptcy indicated by enterprises, is fairly interesting. In variant 1 (Y1 $\le$ alpha1), corresponding to the smallest decrease in orders, the coefficient is negatively related to those enterprises that did not suffer much because of the pandemic and is not threatened by bankruptcy. In variants 2 and 3, the same parameter estimates have positive signs increasing vulnerability for the crisis consequences.

Looking at differences related to the medium-sized enterprises, the presence of X8 in the first variant is to be mentioned. This factor indicates that the deferring leasing installments were efficiently applied in this group of enterprises, although it was not helpful in decreasing transport orders. In this group absence of variables, X11 and X15 are essential. It means that medium enterprises did not suffer significantly from the decrease in profitability (X11). They were not affecting much by the aid offered by the government (X15).

As concerns micro and small enterprises, the variables X15 and X18 are present only in the third variant, i.e., enterprises mostly affecting by consequences of the crisis. What is essential, the impact of the size of these enterprises was strongly confirmed. It was represented by two variables, X29 (the number of transport units) and X32 (the number of employees). Both were acting as factors of enterprises’ resilience across all variants of answers.

## Discussion

Summing up, the road transport enterprises in Poland were vulnerable to the crisis caused by the pandemic in both financial aspects, as defined by the IMF, and logistics as defined by Barnes and Oloruntoba (2005). The crucial factor of enterprises' resilience to the crisis was related to low fuel prices during the first phase of the pandemic. Medium-sized enterprises were more resilient than the micro and small ones, as they were able to manage their resources and customer relations more efficiently in the first phase of the unforeseen crisis. Micro and small enterprises benefited from the government's anti-crisis policy and direct aid coming from the state. That is why, the number of trucks and the number of employees occurred significantly in the first phase of the crisis as a factor of resilience. It can be explained that the aid was first directed to micro and small businesses.

Although a similar analysis was not published elsewhere, the results confirm a generally negative impact of the crisis pandemic. As it was mentioned in the introduction, the pandemic had negative consequences for micro and small enterprises in many regions (the USA—Senz 2020; Pakistan—Shafi et al. 2020). Osińska and Zalewski (2020) established that Polish road transport enterprises widely used anti-crisis crisis policy tools directed to the enterprises between April and June 2020. Micro and small enterprises were the first beneficiaries of the aid. The scale of changes in Polish road transport enterprises' situation can be compared to the study reported by Zalewski (2019). At the beginning of 2019, more than 60% of respondents were not afraid of a decrease in the number of transport orders or contractors' loss. The most significant concerns related to changes in the law in the European Union and problems in financing the activity. Up to 52% of respondents in the cited study did not have any integrated Transport Management System, and over 81% did not employ a person dedicated to analyzing transport's financial and market situation. A current study indicates that using innovative IT solutions in road transport increased significantly. Such an innovative attitude was enforced by necessity, like electronic documentation of transactions, looking for cost reduction and distance policy, but it created a new quality in the transport industry.

Apart from the results derived based on the ordered logit models presented in section ``Vulnerability and resilience of transport enterprises–the results from empirical ordered logit models'', also the qualitative analysis presented in section ``The road transport industry in Poland under the pandemic restrictions''  gave some implications for generalization. Firstly, it was confirmed that road transport companies started to use the IT technology in a broad range. On one side it was enabled by the administration authorities who accepted digital documents from both enterprises and drivers, and changed the way of operating of the road transport enterprises. This is in line with the IRU Annual Report (IRU 2021a). As it comes from the literature review also reduction in gas emissions indicated that road transport has a heavy impact on air pollution. There is solid evidence to assume that it will result in the innovative change of the transport units and wide use of the eco-trucks (IRU 2021a).

The mentioned facts strongly support the concept of creative destruction by providing evidence of the innovative change in the road transport industry. Also, Foster and Kaplan (2001) turned the attention to the universality and flexibility of the IT systems and the entire information management as impacting the company’s cost and creating its development goals. The report issued by UNECE (UNECE 2021) supports the viewpoint that Intelligent Transport Systems (ITS) have the potential to revolutionize mobility, to design transport legislation and vehicles regulation. A broad perspective for both the company’s goals and its environment is essential for effective management and creating a sustainable transport system.

The respondents were asked whether the pandemic state forced a change of existing contractors and industries served in the survey. Only 16.4% of respondents indicated the need to look for new contacts, and 13.2% gave no precise answer to the question. This information should be considered together with the economic sectors to which transport services are assigned. As Ivanov and Desgui (2020) showed, there was huge disequilibrium in the reaction of particular economy sectors for the pandemic state. Some sectors suffered from excess demand, and the others were subject to insufficient orders. This observation brought the authors to the concept of a novel decision-making environment that considers supply networks and viability as integrity to ensure survivability at a large scale. Besides, in the scale of the transport enterprise, it is also worth considering portfolios of orders diversification according to the economic sector as well as domestic and international transportation. Furthermore, being included in a supply chain (or network), transport enterprise managers should realize how flexible and efficient the supply chain is flexible and efficient in the case of unpredicted shocks.

Rothengatter (2011) analyzed the short and medium-term innovations that helped the road transport companies to survive after the global financial crisis. The current crisis, caused by the COVID-19 pandemic, enabled us to uncover several positive changes in the short and medium run, such as a wide application of IT, documents digitalization, lower gas emissions which are likely to change road transport in a sustainable direction.

## Conclusions

The research aimed to examine the vulnerability and resilience of road transport enterprises in Poland—especially in international transport—for the economic crisis that resulted from the first phase of the COVID-19 pandemic. The selection of the first phase was motivated by the unpredicted shock that accompanied the first lockdown. The following waves – although highly negative – were possible to be predicted.

The empirical analysis, based on survey data, made in July 2020 on 500 road transport enterprises showed that this specific branch, which is very sensitive for changes in global trade, suffered much from the COVID-19 pandemic because:

- The lockdown, which in Europe started on March 10, 2020, resulted in border crossing limitations and sanitary restrictions.
- Demand for transport services, measured as the number of transport orders, decreased significantly due to production and trade limitations.
- The prices of freight per 1 km were lower.

Additionally, costs in the transport industry are increased by leasing installments for using the newest generations of vehicles. These reasons caused transport enterprises, which mostly belong to micro, small and medium enterprises, were imposed on the risk of bankruptcy. The negative phenomena in the transport market were accompanied by the positive—for costs reduction – changes observed on the fuel and currency markets. Global prices of fuel fell due to lower global demand. Polish currency PLN was depreciated against the euro. However, these factors could not fully mitigate decreased demand, which was observed in most sectors of the economy. Therefore, road transport enterprises widely used the anti-crisis aid of the Polish government.

We estimated partial proportional odds models ordered logit model to identify factors responsible for the enterprises' vulnerability and resilience on the pandemic's unforeseen demand shock. A set of statistical indicators and tests was performed to justify the results. It allows the division of enterprises according to the level of vulnerability for the lowered demand. Three empirical models were estimated for three subsamples: total number of enterprises, medium enterprises, and micro and small enterprises. The results show that the decrease in truck fuel prices acted as the main factor of resilience. Its parameter estimate is negative across all vulnerability levels indicated by the partial proportional odds models ordered logit model and all subsamples. Medium-sized enterprises were more resilient than the micro and small ones, while the latter benefited from the government's anti-crisis policy and direct aid coming from the state.

The other factors, such as a decrease in the number of orders, a decrease in profitability, a scale of decrease in profitability, and the government anti-crisis policy implementation, increase the vulnerability of transport enterprises located in Poland to such consequences as a decreasing scale of transport services. We found that the generalized ordered logit model and the resulting partial proportional odds models were useful for identifying vulnerability and resilience factors to a crisis and determining groups of enterprises according to the small, medium, and high levels of exposure.

The results of both estimated models and descriptive analysis of the responses enabled to formulate the recommendations concerning transport activity in Poland in the medium term, which can be adopted worldwide.

Firstly, the logit model shows that enterprises should apply careful cost analysis and lean float management. In general, the managers are conscious of the meaning of costs and quality of transport services, including continuous float improvement in the competitive European market, and the reported study strongly supported its significance. Secondly, the descriptive analysis and literature review confirmed that it is crucial to diversify portfolios of orders and contractors, mainly domestic and international transportation. It can be done at the level of a supply chain or individual enterprise. Thirdly, the flexibility of the structure of the float of vehicles is recommended. It is related to the former recommendation. While huge trucks and vehicles are significant for long distances, it is worth noting that light cars support trade and delivery for short distances, and they can be used supplementary, particularly on domestic routes. Finding an optimum transport float structure is not easy, but it is related to the third recommendation, i.e., managing financial resources, including relations between own and borrowed capital. Noting that transport is most often realized for low freight prices by micro, small and medium enterprises, it is recommended to formulate strategic goals for each size of business in the context of economic and legal constraints. It is essential because the government aid in the crisis period can only be active in the short run.

We defined the COVID19 pandemic as a discontinuity, considered within the Schumpeterian creative destruction concept which should result in a complex insight into transport enterprises' management. The empirical results, both descriptive and quantitative, revealed positive aspects of the COVID-19 pandemic, resulting in the wide IT application as well as gas emission reduction. Both effects can cause innovative and sustainable solutions in transportation. However, it is too early to conclude that the road transport industry in Poland managed with all negative aspects of the current crisis and created an innovative solution for sustainable development. It certainly needs further investigation in the nearest future. Furthermore, the road transport industry is subject to change due to legal and environmental requirements, market needs, and supply chain connections. Increased quality of the services supported by innovative IT solutions, precise analytics, and transportation sustainability becomes an important factor in the competition in the European market. A system of innovative solutions based on mobile service operations, obviously accelerated by the COVID-19 pandemic, has been proposed and discussed by Choi (2020).

## Data Availability

Data are available from the authors on request.

## Footnotes

1 One should take into account “normal” frictions in registers.

2 https://coronavirus.jhu.edu/map.html (accessed 10.08.2021).
